# Computed tomography-based 3D convolutional neural network deep learning model for predicting micropapillary or solid growth pattern of invasive lung adenocarcinoma

**DOI:** 10.1007/s11547-024-01800-3

**Published:** 2024-03-21

**Authors:** Jiwen Huo, Xuhong Min, Tianyou Luo, Fajin Lv, Yibo Feng, Qianrui Fan, Dawei Wang, Dongchun Ma, Qi Li

**Affiliations:** 1 Department of Radiology, the First Affiliated Hospital of Chongqing Medical University, No. 1 Youyi Road, Yu Zhong District, Chongqing, 400016 China; 2 Anhui Chest Hospital, 397 Jixi Road, Hefei, 230022 Anhui Province China; 3 Institute of Research, Infervision Medical Technology Co., Ltd, 25F Building E, Yuanyang International Center, Chaoyang District, Beijing, 100025 China

**Keywords:** Lung cancer, Adenocarcinoma, Tomography, X‑ray computed, Deep learning, Pathology

## Abstract

**Purpose:**

To investigate the value of a computed tomography (CT)-based deep learning (DL) model to predict the presence of micropapillary or solid (M/S) growth pattern in invasive lung adenocarcinoma (ILADC).

**Materials and Methods:**

From June 2019 to October 2022, 617 patients with ILADC who underwent preoperative chest CT scans in our institution were randomly placed into training and internal validation sets in a 4:1 ratio, and 353 patients with ILADC from another institution were included as an external validation set. Then, a self-paced learning (SPL) 3D Net was used to establish two DL models: model 1 was used to predict the M/S growth pattern in ILADC, and model 2 was used to predict that pattern in ≤ 2-cm-diameter ILADC.

**Results:**

For model 1, the training cohort’s area under the curve (AUC), accuracy, recall, precision, and F1-score were 0.924, 0.845, 0.851, 0.842, and 0.843; the internal validation cohort’s were 0.807, 0.744, 0.756, 0.750, and 0.743; and the external validation cohort’s were 0.857, 0.805, 0.804, 0.806, and 0.804, respectively. For model 2, the training cohort’s AUC, accuracy, recall, precision, and F1-score were 0.946, 0.858, 0.881,0.844, and 0.851; the internal validation cohort’s were 0.869, 0.809, 0.786, 0.794, and 0.790; and the external validation cohort’s were 0.831, 0.792, 0.789, 0.790, and 0.790, respectively. The SPL 3D Net model performed better than the ResNet34, ResNet50, ResNeXt50, and DenseNet121 models.

**Conclusion:**

The CT-based DL model performed well as a noninvasive screening tool capable of reliably detecting and distinguishing the subtypes of ILADC, even in small-sized tumors.

## Introduction

Lung cancer is one of the most common cancers and the major cause of cancer-related deaths worldwide, with lung adenocarcinoma (LADC) being the most prevalent histological type [1]. According to the Classification of Lung Tumors, 5th edition, by the World Health Organization (WHO) in 2021, LADC can be classified into the invasive type and microinvasive type [2]. The most common type is invasive adenocarcinoma (ILADC), which mainly manifests as lepidic, acinar, papillary, micropapillary, and solid growth patterns [3]. Currently, the clinical treatment of ILADC is mainly based on 9th edition of TNM staging system [4], assessing tumor size, lymph nodes, and metastasis, with treatments ranging from surgery in early stages (I–II) to chemotherapy and radiotherapy for advanced cases (III–IV). Extensive research has demonstrated that ILADC has a significantly higher recurrence and metastasis hazard with a micropapillary or solid-predominant growth pattern than without [5–7]. Notably, some research has indicated that a micropapillary or solid growth pattern (M/S pattern) > 5% is also associated with poor outcome of patients with ILADC [6–10], and further aggressive adjuvant treatment and dissection or sampling of lymph nodes during surgery are usually recommended [7, 11, 12]. In addition, some recent studies have recommended that segmentectomy become the standard surgical procedure for patients with small-sized (diameter ≤ 2 cm) non-small-cell lung cancer (NSCLC), but limited resection was not the optimal surgical approach for patients with M/S-pattern tumors [5, 13, 14]. Therefore, preoperative confirmation of an M/S pattern within ILADC is significantly important to determine the resection range and guide surgical planning. Generally, surgery is the most effective technique to evaluate the histological subtypes of ILADC. However, to date, few operative methods can recognize the M/S pattern before or during surgical resection. A new noninvasive method to identify the histological subtypes of ILADC before surgery could be a valid tool to reduce the occurrence of inappropriate surgical plan choices.

Deep learning (DL) is a general term for a class of pattern analysis methods that typically include convolutional neural network (CNN), deep belief network, and stacked auto-encoder network [15]. With the development of artificial intelligence, DL has been widely used in the early diagnosis of lung cancer, evaluation of tumors’ pathological-molecular characteristics, and prediction of patients’ outcomes [16, 17]. Regarding estimating the histological subtypes of ILADC by artificial intelligence, previous studies were either limited in their ability to discriminate between the predominant growth patterns of ILADC or concentrated on patients in advanced stages [18, 19]. To our knowledge, the capacity of DL to predict the presence of an M/S growth pattern of ILADC, particularly in small-sized tumors, has still not been investigated.

This study aimed to develop two CT-based DL models: one for predicting the M/S growth pattern in ILADC, and the other for predicting that pattern in ≤ 2-cm-diameter ILADC.

## Materials and methods

### Patients

**Fig. 1:**
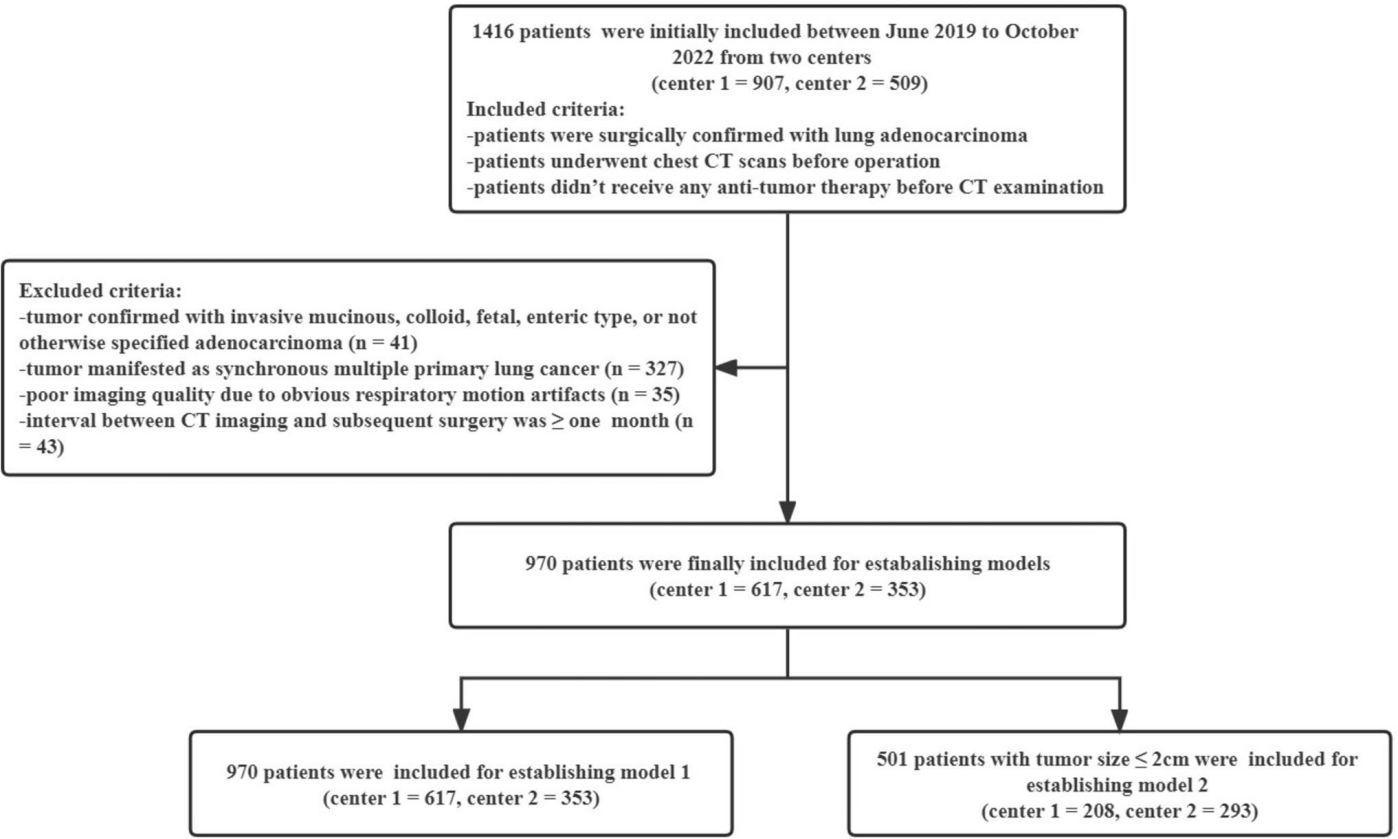
Patient inclusion flowchart

This retrospective study was approved by the institutional ethics committee of our institution (approval number: 2019-062), and informed consent for research participation was waived due to the retrospective nature. From June 2019 to October 2022, 1416 patients from two centers (center 1 = 907, center 2 = 509) were initially included according to the following inclusion criteria: (1) ILADC was surgically confirmed; (2) patients had undergone chest CT scans before operation; and (3) patients had not undergone any anti-tumor therapy before CT examination. Additionally, 446 patients were excluded according to the following exclusion criteria: (1) tumor confirmed with invasive mucinous adenocarcinoma, colloid adenocarcinoma, fetal adenocarcinoma, enteric type adenocarcinoma, or not otherwise specified (*n* = 41); (2) tumor manifested as synchronous multiple primary lung cancer (*n* = 327); (3) poor imaging quality due to obvious respiratory motion artifacts (*n* = 35); (4) ≥ 1-month interval between CT imaging and subsequent surgery (*n* = 43). Finally, the CT data of 970 patients (center 1 = 617, center 2 = 353) were used to build a DL model (model 1). Additionally, the CT data of 501 patients (center 1 = 208, center 2 = 293) with tumor diameters ≤ 2 cm were used to establish another DL model (model 2). Patients from center 1 were divided into the training and internal validation datasets in a ratio of 4:1, and those from center 2 were included as the external validation dataset. The patient inclusion flowchart is shown in Fig. 1.

### CT protocols

All patients underwent chest CT scans using one of the following CT systems: Discovery 750 HD CT (GE Healthcare, Milwaukee, WI, USA), Somatom Perspective (GE Healthcare, Erlangen, Germany), or Somatom Definition FLASH (Siemens Healthcare, Forchheim, Germany). During a single breath-hold period, the CT scan was performed at the end of inspiration. The scan range was from the entrance of the thorax to the costophrenic angle. The scanning parameters were as follows: tube voltage, 100–130 kVp; automatic tube current, 50–250 mAs, scanning slice thickness/interval, 5 mm/5 mm, and reconstructed thickness/interval, 0.625–1 mm/0.625–1 mm. Then, all images were transferred to the picture archiving and communication system workstation.

### Histochemical examination

Histological samples were obtained from surgical resection. All selected specimens stained with hematoxylin and eosin were analyzed by an experienced pathologist. According to the current LADC classification system, the percentage of some growth patterns (lepidic, acinar, papillary, micropapillary, solid) in the tumor with > 5% increments were considered to be indicative of this pattern.

### Data preprocessing

All CT images in Digital Imaging and Communications in Medicine (DICOM) format were imported into the Infer Scholar Center platform (https://www.infervision.com/, Infer Scholar). Lesion region of interest (ROI) was initially manually denoted with bounding boxes slice by slice on axial CT images by a radiologist with 5 years of chest imaging experience, then that was reviewed and corrected by a radiologist with more than 10 years of chest imaging experience for accuracy. All the CT images were evaluated in a standard lung window setting {window width: 1500–2000 HU, window level: − 500 to (− 700) HU}to ensure optimal image quality and detail for accurate analysis. Thereafter, CT images were processed as follows: first, every 2D DICOM slice was concatenated to a 3D pixel matrix. Given that different image spacing will affect the recognition accuracy of 3D CNNs, the 3D pixel matrixes were resampled to obtain a 0.5 mm × 0.5 mm × 0.5 mm (height × width × depth) resolution with linear interpolation, and the pixel values were clipped to obtain lung windows (1400 HU, 200 HU). Second, all input images were normalized and padded to the same size. After acquiring these images, we randomly cropped them to 128 × 128 × 128 pixels and then performed random Gaussian noise, rotation, scaling, and flipping to reduce overfitting. Third, the augmented image patches were fed into the classification model for training. In the training process, to distinguish hard samples in the training set, we used self-paced learning (SPL), which can gradually incorporate hard samples into training. The initial threshold, threshold growth rate, and start epoch of SPL were 0.7, 1.05, and 20, respectively.

### DL models establishment and optimization

**Fig. 2:**
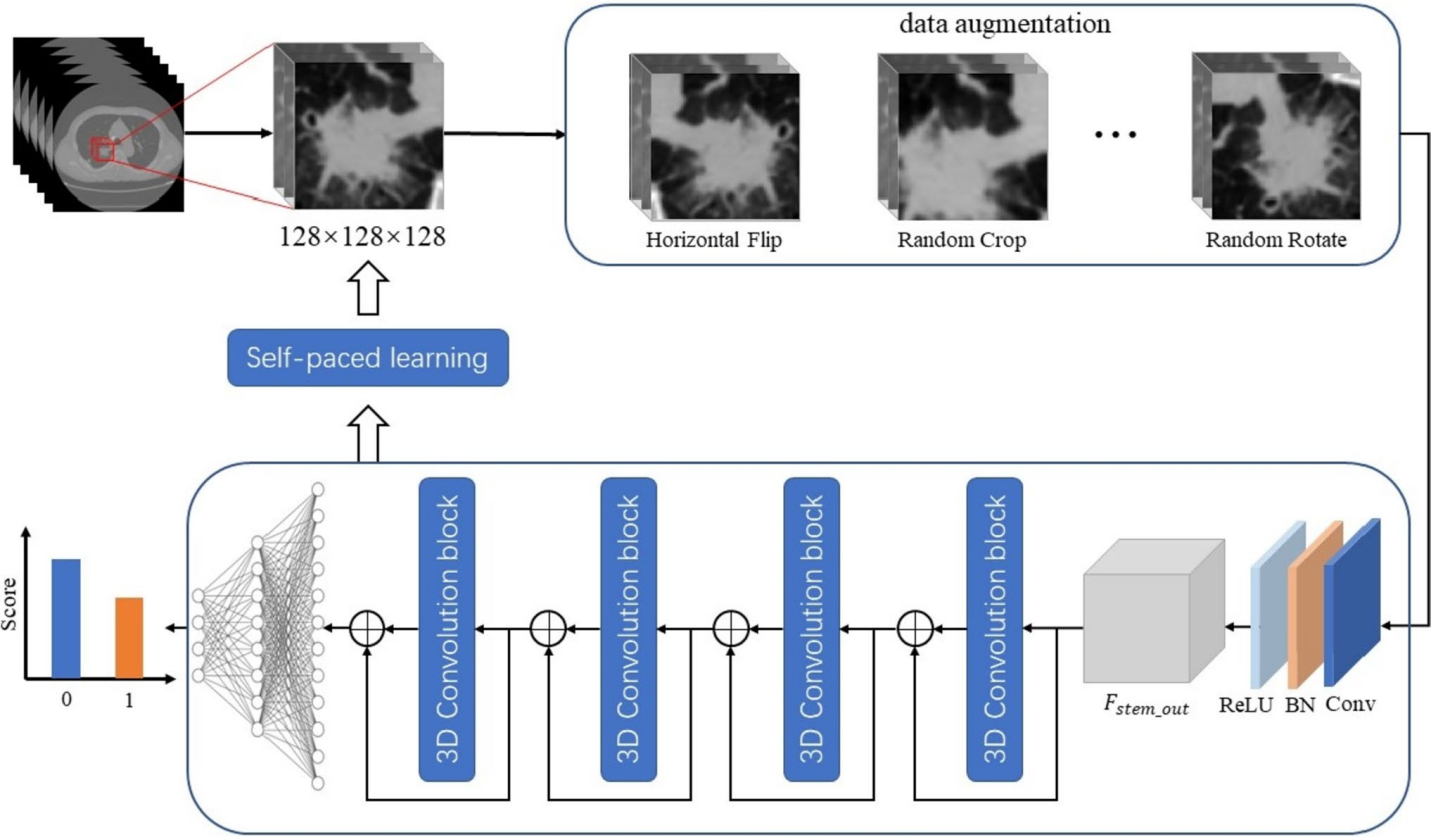
Workflow for model establishment

Two DL models were established in this study. Model 1 was used to predict the presence of M/S pattern in ILADC, and model 2 was used to predict the presence of M/S pattern in small-sized ILADC, which was defined as a tumor with a maximum diameter ≤ 2 cm in the lung window setting. Thereafter, 3D ResNet18 was adapted to build models, which included four-feature extraction stages and one classification stage. The number of output channels in the four-feature extraction stages were 64, 128, 256, and 512, respectively. The feature maps generated in the last feature-extraction stage were fed into the fully-connected layer to predict the probability of presence of M/S pattern. Adam W and cross entropy loss were used to optimize the classification models. The training batch size, learning rate, and epochs were 16, 0.0005, and 200, respectively. The workflow for model establishment, is shown in Fig. 2. The area under the curve (AUC), accuracy, recall, precision, and F1-score were used to evaluate the performance of both models. Additionally, another four DL networks, including ResNet34, ResNet50, ResNeXt50, and DenseNet121, were used to build models, and their performances were compared with those of 3D ResNet18.

### Statistical analysis

Two GTX 3090 graphics-processing units were used. The operating system was Ubuntu 20.04 with CUDA, version 11.3. Python 3.7 with PyTorch 1.11.0 were used to implement the models. Statistical analyses were performed with the SPSS 25.0 software package (version 25.0; IBM SPSS Statistics for Windows, IBM Corp., Armonk, NY, USA). The independent sample *t*-test was used to evaluate age, which was normally distributed, and *P* < 0.05 was considered to be indicative of a statistically significant difference.

## Results

### Demographic and clinical characteristics

The demographic and clinical characteristics of the patients included in the databases for building the DL models are summarized in Table 1. A total of 970 patients (454 men and 516 women) were classified into the database for establishing model 1, including 492, 125, and 353 patients in the training, internal validation, and external validation cohorts, respectively, with a mean age of 60.2 ± 9.7 years (range: 25–85 years). Among them, 510 patients were in pathological tumor–node–metastasis (pTNM) stage I, 317 were in stage II, and 143 were in stage III. A total of 501 patients (235 men and 266 women) in the database were used to establish model 2, including 161, 47, and 293 patients in the training, internal validation, and external validation cohorts, respectively, with a mean age of 58.9 ± 9.9 year (range: 25–83 years). Among them, 315 patients were in pTNM stage I, 155 were in stage II, and 31 were in stage III.

**Table 1:** Demographic and clinical characteristics of the patients

Characteristics	Model 1 (*n* = 970)			Model 2 (*n* = 501)		
	Training cohort (*n* = 492)	Internal validation cohort (*n* = 125)	External validation cohort (*n* = 353)	Training cohort (*n* = 161)	Internal validation cohort (*n* = 47)	External validation cohort (*n* = 293)
Mean age (years)	60.8 ± 9.8	60.8 ± 9.3	59.1 ± 9.6	59.5 ± 10.2	58.7 ± 10.4	58.6 ± 9.7
Sex						
Female	270 (54.9%)	65 (52.0%)	181 (51.3%)	92 (57.1%)	18 (38.3%)	156 (53.2%)
Male	222 (45.1%)	60 (48.0%)	172 (48.7%)	69 (42.9%)	29 (61.7%)	137 (46.8%)
Histological subtypes						
Presence of M/S	205 (41.7%)	52 (41.6%)	174 (49.3%)	54 (33.5%)	16 (34.0%)	133 (45.4%)
Absence of M/S	287 (58.3%)	73 (58.4%)	179 (50.7%)	107 (66.5%)	31 (66.0%)	160 (54.6%)
pTNM stage						
I	254 (51.6%)	64 (51.2%)	192 (54.4%)	108 (67.1%)	27 (57.4%)	180 (61.4%)
II	163 (33.1%)	42 (33.6%)	112 (31.7%)	42 (26.1%)	16 (34.0%)	97 (33.1%)
III A	75 (15.2%)	19 (15.2%)	49 (13.9%)	11 (6.8%)	4 (8.5%)	16 (5.5%)

*M/S*  micropapillary or solid growth pattern, *pTNM*  pathological tumor–node–metastasis

### Performance of Model 1 and Model 2 built by SPL 3D Net

For model 1 established by SPL 3D Net, the AUC, accuracy, recall, precision, and F1-score in the training cohort were 0.924, 0.845, 0.851,0.842, and 0.843 respectively; those in the internal validation cohort were 0.807, 0.744, 0.756, 0.750, and 0.743, respectively; and those in the external validation cohort were 0.857, 0.805, 0.804, 0.806, and 0.804, respectively (Table 2). For model 2 established by SPL 3D Net, the AUC, accuracy, recall, precision, and F1-score in the training cohort were 0.946, 0.858, 0.881, 0.844, and 0.851, respectively; those in the internal validation cohort were 0.869, 0.809, 0.786, 0.794, and 0.790, respectively; and those in the external validation cohort were 0.831, 0.792, 0.789, 0.790, and 0.790, respectively (Table 3).

**Table 2:** Predictive performance of model 1 established by different networks

Model 1	AUC	Accuracy	Recall	Precision	F1-score
Training cohort	0.924	0.845	0.851	0.842	0.843
Internal validation cohort					
ResNet34	0.791	0.736	0.744	0.737	0.734
ResNet50	0.784	0.744	0.726	0.740	0.729
ResNeXt50	0.782	0.736	0.744	0.737	0.734
DenseNet121	0.770	0.728	0.745	0.742	0.728
SPL 3D Net	0.807	0.744	0.756	0.750	0.743
External validation cohort					
ResNet34	0.847	0.799	0.799	0.800	0.799
ResNet50	0.794	0.717	0.716	0.717	0.716
ResNeXt50	0.784	0.714	0.713	0.717	0.712
DenseNet121	0.798	0.725	0.724	0.738	0.720
SPL 3D Net	0.857	0.805	0.804	0.806	0.804

*AUC* Area under the curve

**Table 3:** Predictive performance of model 2 established by different networks

Model 2	AUC	Accuracy	Recall	Precision	F1-score
Training cohort	0.946	0.858	0.881	0.844	0.851
Internal validation cohort					
ResNet34	0.851	0.809	0.764	0.796	0.776
ResNet50	0.802	0.787	0.763	0.763	0.763
ResNeXt50	0.837	0.809	0.794	0.786	0.790
DenseNet121	0.804	0.809	0.779	0.789	0.783
SPL 3D Net	0.869	0.809	0.786	0.794	0.790
External validation cohort					
ResNet34	0.730	0.689	0.686	0.687	0.687
ResNet50	0.756	0.717	0.701	0.734	0.700
ResNeXt50	0.792	0.727	0.726	0.725	0.725
DenseNet121	0.753	0.707	0.705	0.704	0.705
SPL 3D Net	0.831	0.792	0.789	0.790	0.790

*AUC* area under the curve

### Comparison of the performance of models built by different DL networks

**Fig. 3:**
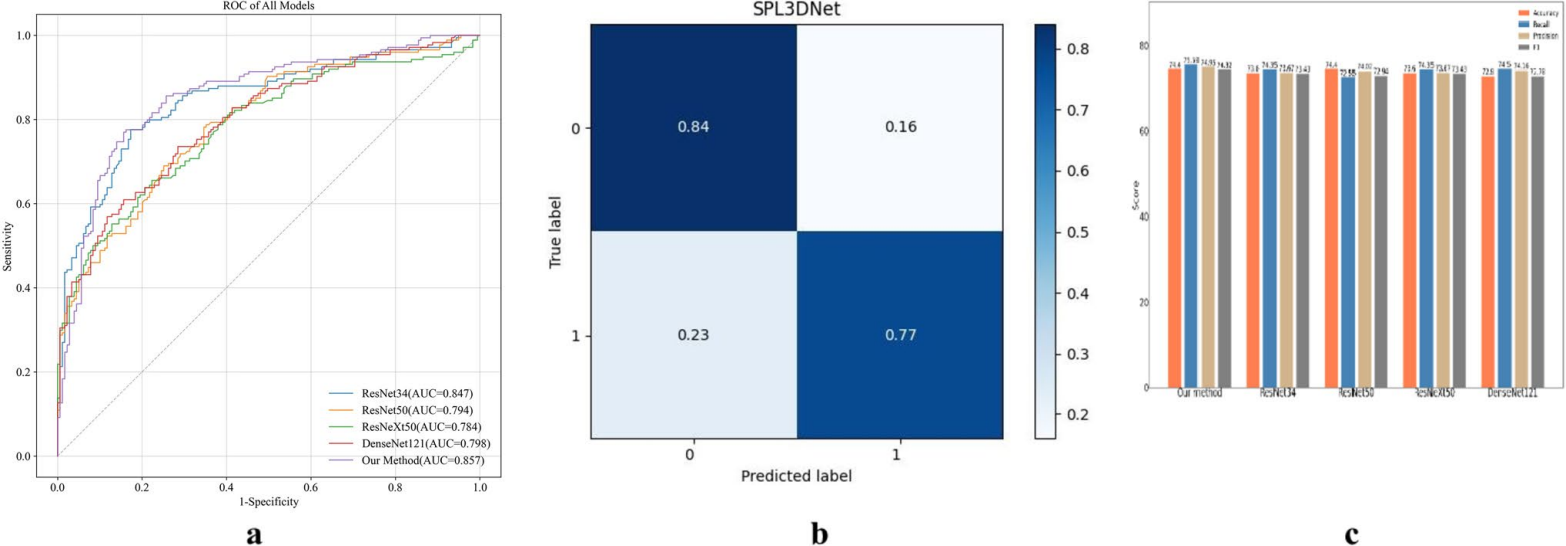
**a** The receiver operating characteristic curves of model 1 established by different networks in the external validation cohort. **b** The confusion matrix of model 1 established by SPL 3D Net in the external validation cohort. **c** Classification performances of model 1 established by different networks in the internal validation cohort

**Fig. 4:**
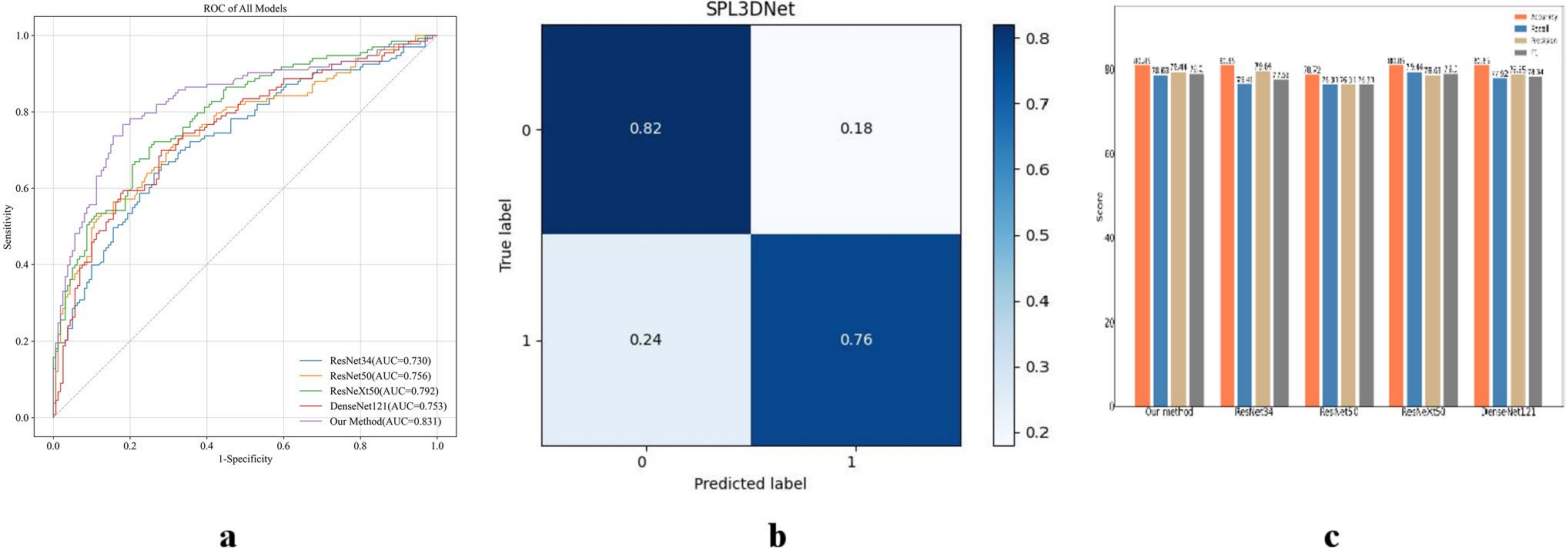
**a** The receiver operating characteristic curves of model 2 established by different networks in the external validation cohort. **b** The confusion matrix of model 2 established by SPL 3D Net in the external validation cohort. **c** Classification performances of model 2 established by different networks in the internal validation cohort

The performance of models built by ResNet34, ResNet50, ResNeXt50, and DenseNet121 with distinct network structures and depths was compared to that of the models built by SPL 3D Net in the internal and external validation cohorts. The ability to predict the presence of an M/S growth pattern of ILADC was better in the models using SPL 3D Net than in the models using the other four DL networks (Figs. 3 and 4).

## Discussion

The histopathological LADC subtype is a critical determinant for the postoperative recurrence and metastasis of tumors. Some studies have shown that the M/S-predominant subtype is an undesirable prognostic factor for patients with resected early-stage LADC, and postoperative adjuvant therapy is usually required [6–8, 10]. Furthermore, a few scholars have indicated that the presence of an M/S component in LADC is also highly suggestive of poor survival [5, 9, 20]. Up to now, many scholars have used omics or DL based on CT images to attempt to noninvasively predict the predominant growth pattern of ILADC before operation [18, 19]. However, little research has focused on identifying the existence of the M/S component within tumors by machine learning. Therefore, we developed and validated a 3D-CNN DL model based on CT images to investigate its potential to predict the presence of aggressive histological subtypes of ILADC and placed an emphasis on tumors with a diameter of ≤ 2 cm.

Recently, a few studies have reported the technical success of artificial intelligence in evaluating the growth patterns of ILADC. He B et al. [21] developed four radiomics-based machine-learning models to preoperatively predict the presence of M/S growth patterns and obtained AUC values of 0.75, 0.73, 0.72, and 0.74 in the internal validation and those of 0.70, 0.72, 0.73, and 0.69 in external validation for Naïve Bayes, support vector machine, random forest, and a generalized linear model, respectively. Choi et al. [19] revealed that a DL model was useful in estimating the presence of ≥ 5% M/S histological patterns of tumor and predicting the clinical outcomes of patients with advanced LADC who underwent neoadjuvant therapy. Chen et al. [22] proposed that radiomics combined with a DL model could be used to predict the presence of high-grade growth patterns within LADC manifesting as subsolid lesions, with an accuracy of 0.966. Consistent with previous research, the current study established a DL model to predict the presence of the M/S component in ILADC, which showed good performance in the training, internal validation, and external validation sets, with AUCs of 92.37, 80.65, and 85.65, respectively. Compared with previous studies, our research has the following advantages: first, our DL model established by SPL 3D Net exhibited better performance in both the training and test sets; second, the sample size in this study was larger, and an external validation cohort was used to test the models’ generalization.

A phase 3 clinical trial (JCOG0802/WJOG4607L) at 70 institutions in Japan reported better overall survival of patients with peripheral small-sized (diameter ≤ 2 cm) NSCLC in the segmentectomy group than in the lobectomy group [13], and this finding suggested that segmentectomy should be applicable for this population of patients. Additionally, a study by Kilic et al. [23] suggested that elderly patients (≥ 75 years old) with stage I NSCLC undergoing anatomical segmentectomy had a local recurrence rate and overall survival similar to those undergoing lobectomy, but their incidence of perioperative complications was much lower (29.5 vs. 50%). However, if the tumor contains the M/S component, indicating a high risk of recurrence and tumor metastasis, these elderly patients may benefit more from lobectomy and dissection of mediastinal lymph node. Therefore, preoperative noninvasive prediction of the existence of the M/S pattern in small-sized LADC is crucial to choose the best clinical treatment strategy [24]. At present, there is no effective method to accurately determine the pathological growth patterns before surgery; thus, we established another DL model by SPL 3D Net to identify the existence of the M/S growth pattern in ILADC with diameters ≤ 2 cm. This model obtained AUCs of 0.946, 0.869, and 0.831in the training, internal, and external validation sets, respectively, thereby showing great potential. Coincidentally, Li et al. [25] retrospectively analyzed patients with pathologically-confirmed LADC of ≤ 2 cm who presented to three hospitals, and their findings showed that a radiomics model based on CT can be applied to predict the presence of a micropapillary pattern in patients with LADC of ≤ 2 cm, with an AUC of 0.81 in the external validation set.

It is generally known that 3D CNN has excellent performance in medical image diagnosis. In this study, we selected four other networks with different structures and depths of convolutional layers to build models: ResNet34, ResNet50, ResNeXt50, and DenseNet121. The effectiveness of each of these models was compared with that of the SPL 3D Net model. Our results indicated that the ability to predict the presence of M/S growth pattern of ILADC in both the training and validation sets was better for the SPL 3D Net than for the other four DL models. SPL allows the model to determine its learning speed according to its own training state [26]. The number of samples used in training is determined by the loss value during the training process. This strategy involves dynamically dividing the sample sets into simple and difficult sets according to a threshold. Different weights are assigned to these sets, and difficult samples are filtered out in the early stages of training. By combining SPL with a 3D CNN, SPL 3D Net can determine the importance of samples during training, which dynamically changes with feedback from the classifier. As the classifier’s performance improves, samples initially considered difficult can be gradually recognized in later training stages. This helps remove the influence of abnormal samples on the model and enhances the model’s generalization and robustness, which may be a good explanation for our results.

This study had several limitations. First, we did not incorporate clinical and CT morphological features, such as the sex of the patients and proportion of solid components of LADC, into the model. Second, the inference process of DL model is difficult to interpret and lacks a logical basis, which may hinder clinicians from using these models in practice. Consequently, additional research using our model is needed.

In conclusion, our findings demonstrated that the CT-based 3D-CNN DL model can potentially serve as a noninvasive screening tool capable of reliably detecting and distinguishing histological subtypes of ILADC, even for tumors with diameters of ≤ 2 cm.

## Abbreviations

LADC: Lung adenocarcinoma
WHO: World Health Organization
ILADC: Invasive lung adenocarcinoma
M/S: Micropapillary or solid
DL: Deep learning
CT: Computed tomography
CNN: Convolutional neural network
NSCLC: Non-small-cell lung cancer
SPL: Self-paced learning (SPL)
pTNM: Pathological tumor–node–metastasis pathology
